# Bone plate fixation ability on the dorsal and lateral sides of a metacarpal shaft transverse fracture

**DOI:** 10.1186/s13018-021-02575-3

**Published:** 2021-07-07

**Authors:** Yung-Cheng Chiu, Cheng-En Hsu, Tsung-Yu Ho, Yen-Nien Ting, Ming-Tzu Tsai, Jui-Ting Hsu

**Affiliations:** 1 School of Medicine, China Medical University, Taichung, 404 Taiwan; 2 Department of Orthopedic Surgery, China Medical University Hospital, Taichung, 404 Taiwan; 3 Department of Orthopedics, Taichung Veterans General Hospital, Taichung, 407 Taiwan; 4 Sports Recreation and Health Management Continuing Studies-Bachelor’s Degree Completion Program, Tunghai University, Taichung, 407 Taiwan; 5 3D Printing Medical Research Center, China Medical University Hospital, Taichung, 404 Taiwan; 6 Department of Biomedical Engineering, Hungkuang University, Taichung, 433 Taiwan, ROC; 7 School of Dentistry, College of Dentistry, China Medical University, 91 Hsueh-Shih Road, Taichung, 40402 Taiwan; 8 Department of Bioinformatics and Medical Engineering, Asia University, Taichung, 413 Taiwan

**Keywords:** Metacarpal shaft fracture, Bone plate, Dorsal side, Lateral side

## Abstract

**Background:**

Metacarpal shaft fractures are a common hand trauma. The current surgical fixation options for such fractures include percutaneous Kirschner wire pinning and nonlocking and locking plate fixation. Although bone plate fixation, compared with Kirschner wire pinning, has superior fixation ability, a consensus has not been reached on whether the bone plate is better placed on the dorsal or lateral side.

**Objective:**

The purpose of this study was to evaluate the fixation of locking and regular bone plates on the dorsal and lateral sides of a metacarpal shaft fracture.

**Materials and methods:**

Thirty-five artificial metacarpal bones were used in the experiment. Metacarpal shaft fractures were created using a saw blade, which were then treated with four types of fixation as follows: (1) a locking plate with four locking bicortical screws on the dorsal side (LP_D); (2) a locking plate with four locking bicortical screws on the lateral side (LP_L); (3) a regular plate with four regular bicortical screws on the dorsal side (RP_D); (4) a regular plate with four regular bicortical screws on the lateral side (RP_D); and (5) two K-wires (KWs). All specimens were tested through cantilever bending tests on a material testing system. The maximum fracture force and stiffness of the five fixation types were determined based on the force–displacement data. The maximum fracture force and stiffness of the specimens with metacarpal shaft fractures were first analyzed using one-way analysis of variance and Tukey’s test.

**Results:**

The maximum fracture force results of the five types of metacarpal shaft fracture were as follows: LP_D group (230.1 ± 22.8 N, mean ± SD) ≅ RP_D group (228.2 ± 13.4 N) > KW group (94.0 ± 17.4 N) > LP_L group (59.0 ± 7.9 N) ≅ RP_L group (44.5 ± 3.4 N). In addition, the stiffness results of the five types of metacarpal shaft fracture were as follows: LP_D group (68.7 ± 14.0 N/mm) > RP_D group (54.9 ± 3.2 N/mm) > KW group (20.7 ± 5.8 N/mm) ≅ LP_L group (10.6 ± 1.7 N/mm) ≅ RP_L group (9.4 ± 1.2 N/mm).

**Conclusion:**

According to our results**,** the mechanical strength offered by lateral plate fixation of a metacarpal shaft fracture is so low that even KW fixation can offer relatively superior mechanical strength; this is regardless of whether a locking or nonlocking plate is used for lateral plate fixation. Such fixation can reduce the probability of extensor tendon adhesion. Nevertheless, our results indicated that when lateral plate fixation is used for fixating a metacarpal shaft fracture in a clinical setting, whether the mechanical strength offered by such fixation would be strong enough to support bone union remains questionable.

## Introduction

Metacarpal shaft fractures are a common hand trauma [1]. The incidence rate of such fractures is approximately 36–42% [2]. Among the different types of metacarpal fracture, fracture of the metacarpal neck has the highest incidence rate, followed by fracture of the metacarpal shaft; the ratio of metacarpal neck to shaft fractures is approximately 1:2 [3]. Stable metacarpal fractures can be treated through closed means and engagement in early range of motion [4]. Among the various metacarpal shaft fracture patterns, transverse fractures often require surgical fixation to achieve bone union given the small bone contact area of transverse fractures. If proper anatomical reduction is not achieved for a metacarpal fracture, the generation of scissor deformity in the patient would result in the sequela of loss of the hand’s prehension function. Additionally, strong fixation allows the patient to engage in early range of motion, which reduces the possibility of metacarpophalangeal joint stiffness [5].

Currently, several surgical options exist for fixating metacarpal fractures in a clinical setting, namely, (1) Kirschner-wire (K-wire) fixation; (2) regular plate fixation; and (3) locking plate fixation. However, no consensus currently exists on the optimal method for metacarpal fracture fixation. Although K-wire fixation is a minimally invasive fixation method, its stability has been questioned. K-wire fixation may also lead to complications such as K-wire breakage, loss of fracture reduction, and pin tract infection [6–8]. By contrast, regular bone plate fixation offers superior fixation strength, allowing patients to more quickly engage in early range of motion and begin rehabilitation earlier, thus enabling superior treatment results [5]. However, regarding the fixation of metacarpal shaft fractures with locking bone plates, no consensus exists on whether the bone plate should be placed on the dorsal or lateral side of the metacarpal shaft. Physicians who advocate for dorsal placing of bone plates believe that lateral plating can produce a tension band effect and in turn allow superior fixation strength to be achieved. However, the disadvantage of dorsal placing is that it easily leads to adhesion of the extensor tendon, which in turn leads to metacarpophalangeal joint stiffness and a limited range of motion in the hand. Nevertheless, clinical observation and feedback have revealed that compared with dorsal plate fixation for phalangeal fractures, the probability of extensor tendon adhesion and joint stiffness occurring in metacarpal fractures is relatively much lower. Therefore, many physicians are more willing to fixate the bone plate on the lateral side while treating metacarpal shaft fractures [9]. Physicians who advocate for lateral placing of bone plates believe that it can reduce the chance of extensor tendon adhesion. From a biomechanical perspective, the optimal level of fixation strength cannot be achieved with lateral placing; nevertheless, the hand skeleton is not required to bear weight like the skeleton of the lower extremity. Thus, the fixation strength necessary for achieving the required mechanical for bone union would not be too high.

Although K-wire fixation is a cheap and commonly used method for fixating metacarpal shaft fractures, relevant studies have revealed that the fixation ability of K-wires is much weaker than that of bone plates. However, fixation with a bone plate to the dorsal side of the metacarpal shaft would lead to problems such as extensor tendon adhesion. Clinically, some orthopedic physicians have attempted to fix shaft fractures by screwing the bone plate into the lateral side of the metacarpal shaft [9]. However, comprehensive biomechanical experiments that explain the fixation ability of lateral plate fixation are currently lacking. Therefore, the current study aimed to use artificial metacarpal bones to examine the fixation ability of locking plates and regular plates in fixating metacarpal shaft fractures, each of which were fixed on the dorsal or lateral side of the metacarpal shaft.

## Materials and methods

### Specimen preparation

This study used artificial 4th generation third metacarpal bones (Sawbones, Vashon, WA, USA) because it would have been highly difficult to obtain an adequate number of real human metacarpal bones of similar bone quality and size. A total of 35 artificial metacarpal bone specimens were used.

### Fixation approaches

**Fig. 1:**
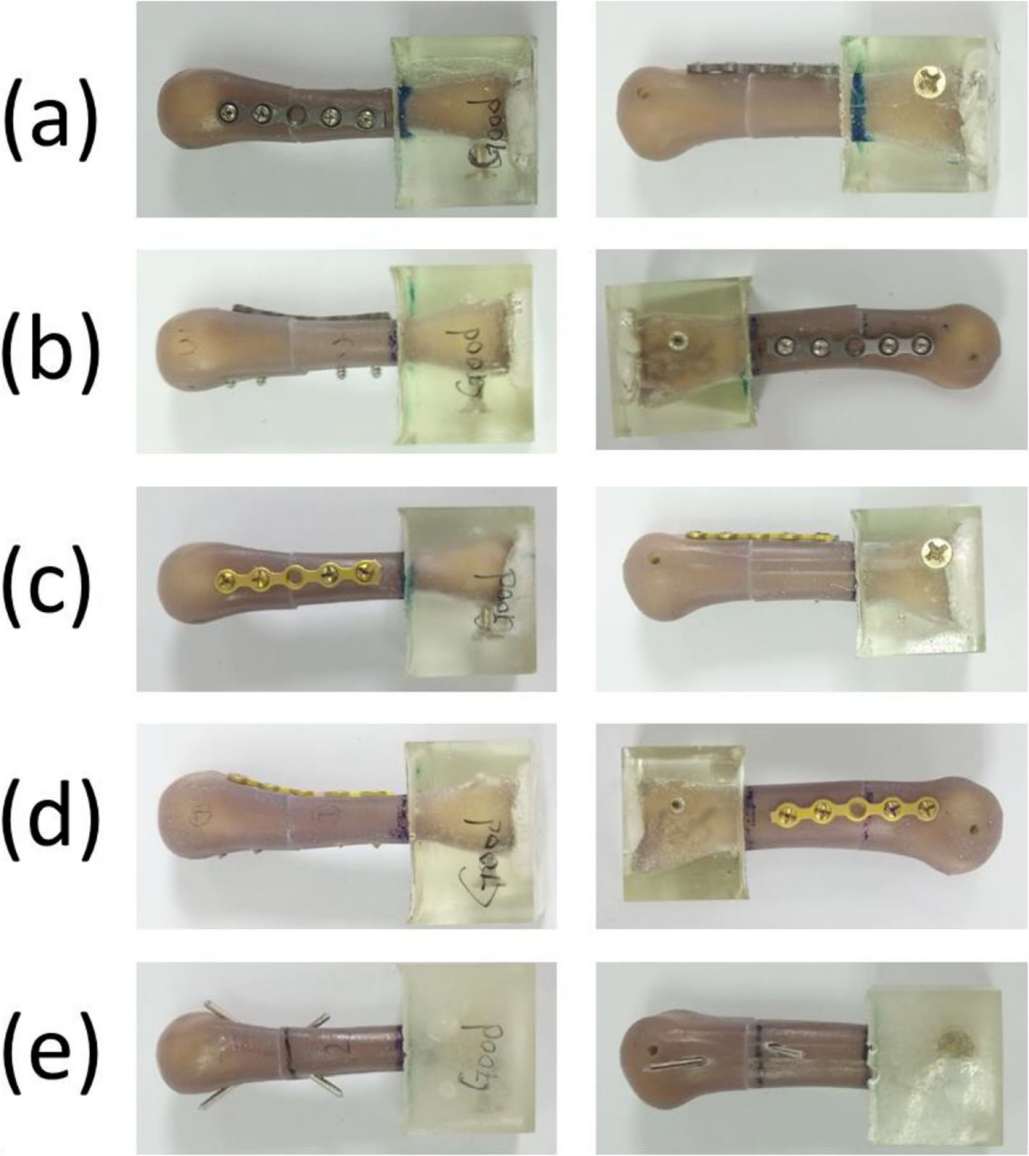
Artificial metacarpal bones and five fixation types for metacarpal shaft fractures: (**a**) locking plate with four locking bicortical screws on the dorsal side; (**b**) locking plate with four locking bicortical screws on the lateral side; (**c**) regular plate with four regular bicortical screws on the dorsal side; (**d**) regular plate with four regular bicortical screws on the lateral side; and (**e**) two K-wires. Left column: anterior–posterior view; right column: lateral view. Note: For clarity, the right columns of (**b**) and (**d**) are the view from the lateral side, and the right columns of (**a**), (**c**), and (**d**) are the view from the medial side

**Fig. 2:**
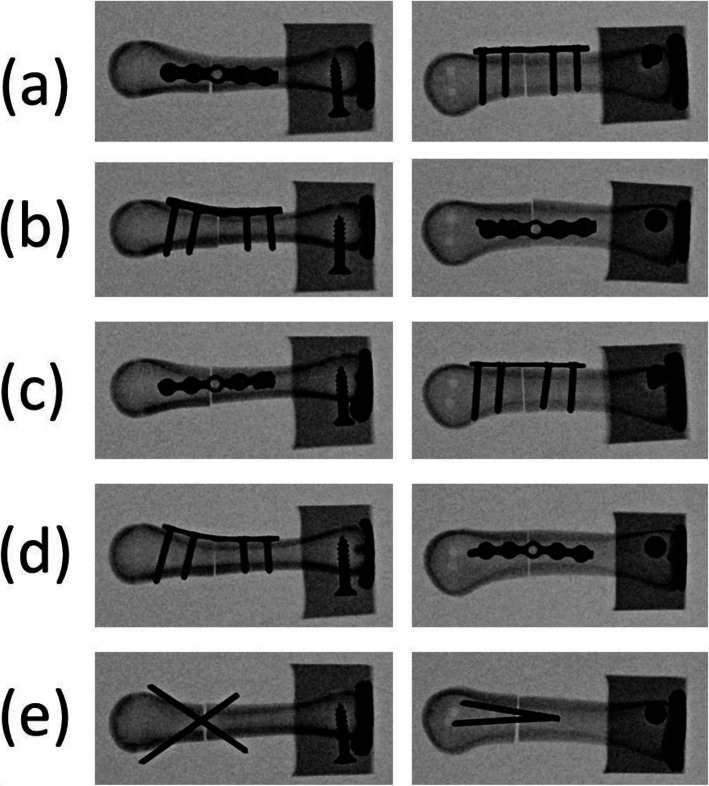
Radiographs of the four fixation types: (**a**) locking plate with four locking bicortical screws on the dorsal side; (**b**) locking plate with four locking bicortical screws on the lateral side; (**c**) regular plate with four regular bicortical screws on the dorsal side; (**d**) regular plate with four regular bicortical screws on the lateral side; and (**e**) two K-wires. Left column: anterior–posterior view; right column: lateral view

Transverse fractures were created in the middle of the artificial third metacarpal bones by using a saw blade. The fracture distance was 30 mm from the distal articular surface. All specimens were assigned to five different fixation techniques performed by single senior hand surgeon (Dr. Yung-Cheng Chiu) (Figs. 1 and 2):

- Group 1—Five-hole locking plate with four locking bicortical screws on the dorsal side (LP_D group): Seven specimens were fixed using a five-hole straight locking plate with locking screws 4 × 2.3 mm in diameter (Stryker, Germany) on the dorsal side of the artificial metacarpal bone. The central hole of the plate was situated on the fracture site, and no screw was fixed. The locking plates were applied at the dorsum of the metacarpal shaft with two bicortical locking screws fixed distally and fixed proximally to the fracture site (Fig. 1a).
- Group 2—Five-hole locking plate with four locking bicortical screws on the lateral side (LP_L group): Seven specimens were fixed using a five-hole straight locking plate with locking screws 4 × 2.3 mm in diameter (Stryker, Germany) on the lateral side of the artificial metacarpal bone. The central hole of the plate was situated on the fracture site, and no screw was fixed. The locking plates were applied on the lateral side of the metacarpal shaft with two bicortical locking screws fixed distally and two fixed proximally to the fracture site (Fig. 1b).
- Group 3—Five-hole regular plate with four regular bicortical screws on the dorsal side (RP_D group): Seven specimens were fixed using a five-hole straight-shape nonlocking plate with compression screws 4 × 2.3 mm in diameter (Stryker, Germany) on the dorsal side of the artificial metacarpal bone. The central hole of the plate was situated on the fracture site, and no screw was fixed. The nonlocking plates were applied at the dorsum of the metacarpal shaft with two bicortical compression screws fixed distally and two fixed proximally to the fracture site (Fig. 1c).
- Group 4—Five-hole regular plate with four regular bicortical screws on the lateral side (RP_L group): Seven specimens were fixed using a five-hole straight nonlocking plate with compression screws 4 × 2.3 mm in diameter (Stryker, Germany) on the lateral side of the artificial metacarpal bone. The central hole of the plate was situated on the fracture site, and no screw was fixed. The locking plates were applied on the lateral side of the metacarpal shaft with two bicortical compression screws fixed distally and two fixed proximally to the fracture site (Fig. 1d).
- Group 5—Two K-wires (KW group): Seven specimens were stabilized with two K-wires 1.5 mm in diameter that were inserted distally from the dorsal medial and lateral side of the metacarpal head–neck junction, penetrating through the fracture site and proximally puncturing and exiting through the proximal volar cortex in cross-pin fixation fashion. Fracture reduction was maintained with manual axial compression during surgery (Fig. 1e).

### Biomechanical test

**Fig. 3:**
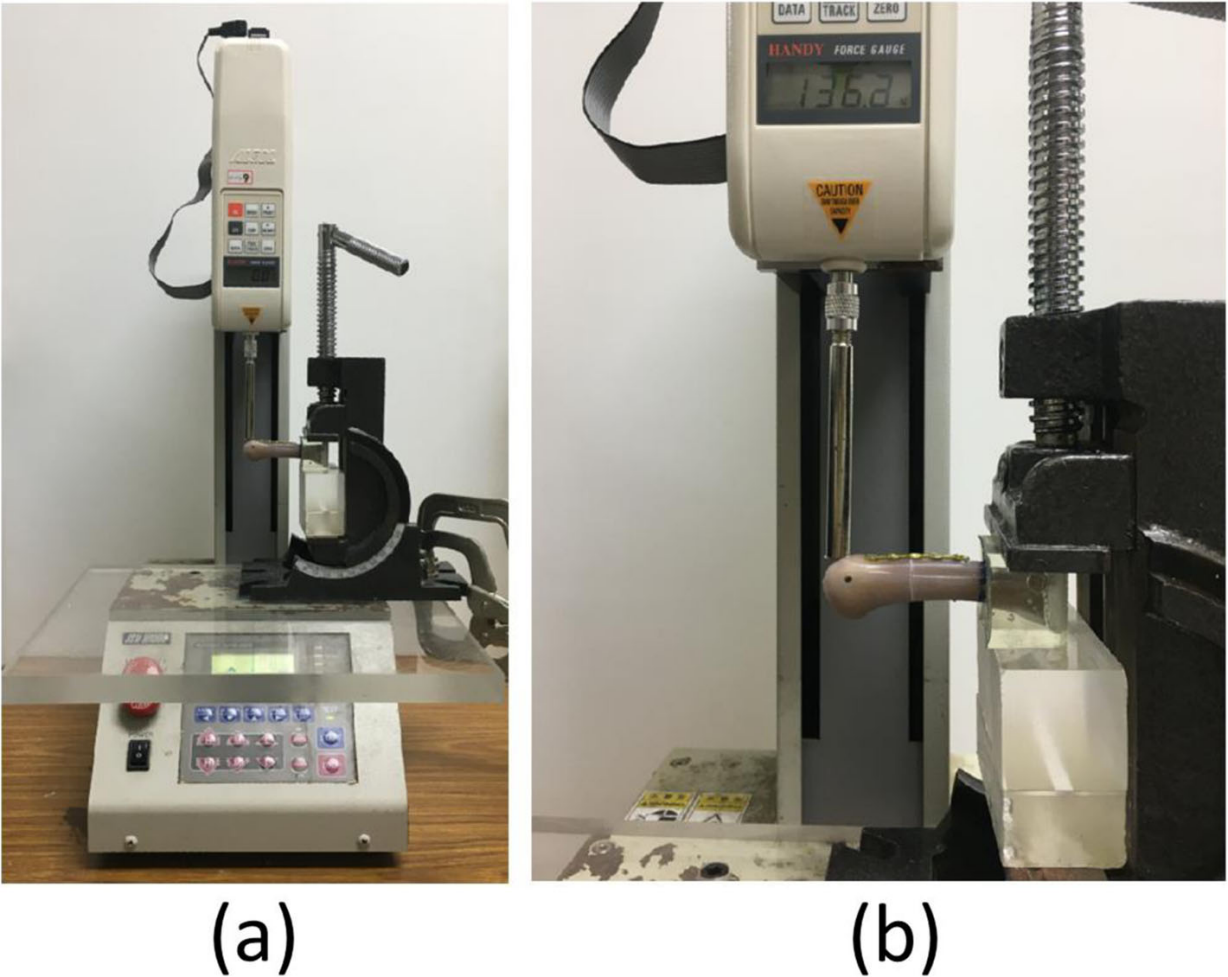
Experimental setup for the cantilever bending test; the specimen is fixated with a regular plate with four regular screws on the dorsal side: (**a**) entire view; (**b**) closed view

Prior to the cantilever beam bending test, the proximal end of each artificial third metacarpal bone was held in a custom fixture using molded epoxy clamps. The tests were conducted using a material testing system (JSV-H1000, Japan Instrumentation System, Nara, Japan; Fig. 3). A perpendicular load was applied to the dorsal side of the specimen at a distance of 50 mm from the fixture until failure. For all tests, the loading speed rate was set as 10 mm/min. The force–displacement data were recorded, and the maximum fracture force and stiffness of each specimen were determined.

### Statistical analysis

The maximum fracture force and stiffness of the specimens with metacarpal shaft fractures and five fixation types were summarized as mean and standard deviation (SD). The maximum fracture force and stiffness of the specimens with fractures were first analyzed using one-way analysis of variance and Tukey’s test, with a 0.05 level of significance. All statistical analyses were performed using SPSS Version 19 (IBM Corporation, Armonk, NY, USA).

## Results

The maximum fracture force and stiffness of the five fixation types are listed in Table 1.

**Table 1:** Maximum fracture force (N) and stiffness (N/mm) of the five fixation types for metacarpal shaft fractures

	Group	Sample size	Mean	SD	MAX	MIN	*P*^a^
Maximum fracture force (N)	LP_D	7	230.1	22.8	263.7	190.4	< 0.001
	LP_L	7	59.0	7.9	70.5	45.0	
	RP_D	7	228.2	13.4	247.3	209.0	
	RP_L	7	44.5	3.4	51.1	38.8	
	KW	7	94.0	17.4	120.4	63.3	
Stiffness (N/mm)	LP_D	7	68.7	14.0	98.2	55.2	< 0.001
	LP_L	7	10.6	1.7	14.6	9.1	
	RP_D	7	54.9	3.2	57.8	48.4	
	RP_L	7	9.4	1.2	11.7	8.3	
	KW	7	20.7	5.8	31.9	15.0	

Groups: *LP_D* locking plate with four locking bicortical screws on the dorsal side, *LP_L* locking plate with four locking bicortical screws on the lateral side, *RP_D* regular plate with four regular bicortical screws on the dorsal side, *RP_L* regular plate with four regular bicortical screws on the lateral side, *KW* two K-wires

^a^One-way analysis of variance

**Fig. 4:**
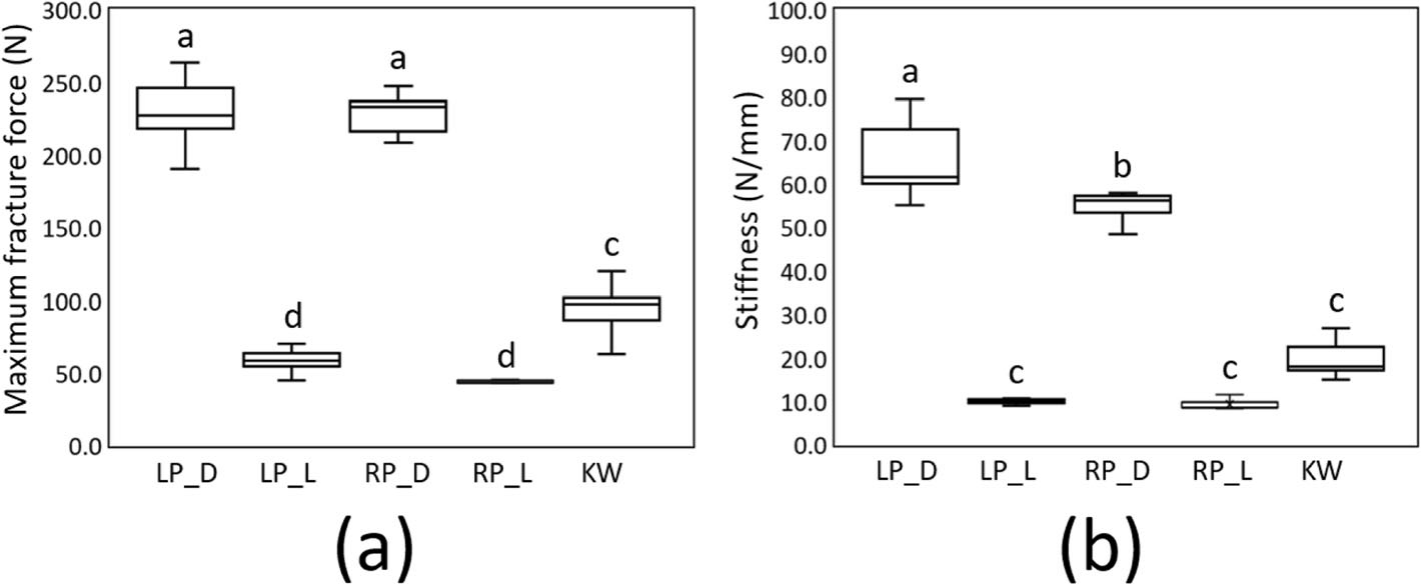
Box plot showing the maximum fracture forces (**a**) and stiffness (**b**) of the five fixation types. Post-hoc pairwise comparisons were conducted using Tukey’s test; the same letters indicate that means were not significantly different at the 0.05 level. LP_D, locking plate with four locking bicortical screws on the dorsal side; LP_L, locking plate with four locking bicortical screws on the lateral side; RP_D, regular plate with four regular bicortical screws on the dorsal side; RP_L, regular plate with four regular bicortical screws on the lateral side; KW, two K-wires

Regarding the maximum fracture force, the highest force among the five fixation types was in the LP_D group, which was 230.1 ± 22.8 N (mean ± SD). The mean of the RP_D group (228.2 ± 13.4 N) was slightly lower than that of the LP_D group (Fig. 4). However, no significant difference was detected between the LP_D and RP_D groups (p = 0.999). The maximum fracture force of both the LP_D and RP_D groups was significantly higher than that of the KW group (94.0 ± 17.4 N; p < 0.001). Furthermore, that of the KW group (94.0 ± 17.4 N) was significantly higher than those of the LP_L (59.0 ± 7.9 N) and RP_L groups (44.5 ± 3.4 N; p < 0.001). In addition, no significant difference was indicated between these LP_L and RP_L groups (p = 0.444; Fig. 4a).

Regarding stiffness, the highest among the five fixation types was in the LP_D group (68.7 ± 14.0 N/mm), which was slightly higher than that of the RP_D group (54.9 ± 3.2 N/mm; p = 0.014). The stiffnesses in the LP_L, RP_L, and KW groups were 10.6 ± 1.7, 9.4 ± 1.2, and 20.7 ± 5.8 N/mm, respectively. However, no significant difference was reported among these three groups (LP_L group vs. RP_L group, p = 0.998; LP_L group vs. KW group, p = 0.120; RP_L group vs. KW group, p = 0.062). The LP_D and RP_D groups had significantly higher stiffnesses than the three lowest three groups (LP_L, RP_L, and KW groups (all p < 0.001; Fig. 4b).

## Discussion

Many hand surgeons support the idea of placing the bone plate on the lateral side of the metacarpal shaft for fracture fixation; doing so, they believe, can reduce the chance of tendon adhesion. In the clinical setting, great success has been achieved by fixating fractures by using this method [10]. Additionally, Shanmugam et al. [11] reported that lateral and dorsal plating did not exhibit mechanical differences in terms of fixating the proximal phalanx. However, comprehensive mechanical experiments that examine the fixation of the metacarpal shaft by using lateral and dorsal plating are currently lacking. Given that metacarpal shaft fractures are a common type of hand fracture, we aimed to verify whether lateral plate fixation can offer sufficient mechanical strength for fracture fixation; we did this through mechanical experiments featuring cantilever bending tests on artificial bones. The experimental results indicated that the dorsal plate fixation of the metacarpal shaft exhibited superior fixation strength—as much as 2.5 times the fixation strength offered by the KW method—regardless of whether a locking or regular plate was used. However, for both types of plates, lateral plate fixation only offered half the fixation strength of the KW method. Lateral plate fixation can reduce the probability of extensor tendon adhesion. Nevertheless, our mechanics research results indicated that when lateral plate fixation is used for fixating metacarpal shaft fractures in a clinical setting, whether the mechanical strength offered by lateral plate fixation would be strong enough to support bone union still remains questionable.

Phalangeal and metacarpal fractures are fairly common, accounting for approximately 10% of all fractures [12]. Metacarpal fractures account for approximately 18% of hand fractures, and the incidence rate is just behind those of distal radius and phalangeal fractures; 70% of metacarpal fractures occur during the second and fifth decades of life, and they are often caused by trauma or sports [13]. Given the populations that are prone to metacarpal fractures, their age, and the hand function loss accompanying such fractures, the losses associated with treatment cost and working time can be tremendous [1, 14, 15]. Most isolated metacarpal fractures can be treated using nonoperative interventions. Relevant cadaveric studies have reported that every 2 mm of shortening would result in approximately 7° of extensor lag [16, 17]. Because most phalangeal joints hyperextend by approximately 20°, the metacarpophalangeal joints can still remain neutral even when the metacarpus is shortened 5 to 6 mm. With regard to angulation in the sagittal plane, be it apex volar or apex dorsal, the angle tolerable by each phalange is different. For example, the phalange for the middle and index finger can tolerate an angle of approximately 10°, that for the ring finger can tolerate an angle of approximately 25°, and that for the pinky finger can tolerate an angle of approximately 45–50° [18, 19]. In fracture deformity, the most intolerable condition is the scissoring deformity caused by malrotation, which occurs during hand prehension: any malrotation deformity larger than 10° would result in clashing or intersection with the adjacent fingers, which would then require surgical intervention [13]. Because of advancements in surgical methods, operative treatments for metacarpal fractures have become increasingly popular over the past decade [20]. The respective fixation strengths of common surgical fracture fixation methods (e.g., dorsal plate, lateral plate, and K-wire fixation) proposed in the current study have a high level of reference value for surgery strategy and rehabilitation exercise planning.

The authors of the current study chose to use artificial metacarpal bones for the experiments given that fresh human metacarpals are difficult to obtain. The study aimed to compare the fixation ability of different fracture fixation methods. Therefore, the authors hoped that each testing sample had almost identical geometrical appearances and material properties. The American Society for Testing and Materials and many related studies have identified artificial bones as being suitable replacements for orthopedic devices as well as for instrument development. Therefore, the researchers used 4th generation artificial metacarpal bones manufactured by Sawbones Corporation; the densities of these artificial cortical bone and cancellous bone are 1.7 and 0.27 g/cm^3^, respectively; said density values closely approximate the density values of real human bones. Additionally, the artificial bones possess a 3.2-mm canal, making them suitable for use as the experimental sample of the current study.

In previous in vitro experiments that have examined the fixation ability for phalangeal or metacarpal fractures, researchers have mainly adopted the following experimental testing methods: (1) cantilever bending tests; (2) modified cantilever bending tests; (3) three-point bending tests; (4) four-point bending tests; (5) and the torsional test. However, these methods are unable to fully simulate physiological conditions commonly found in the clinical setting. Nevertheless, they can be used to evaluate the fixation ability of different fracture fixation methods. The present authors chose to use the commonly applied cantilever bending test as the evaluation method and adopted the maximum fracture force and stiffness as evaluation indicators for evaluating fracture fixation ability. However, as we have explained in our published studies, stiffness represents the rigidity indicator after fracture fixation. Additionally, throughout the duration of the healing process, it is unlikely for such immense active or passive force to occur and lead to the condition of metacarpal bone refracture. Therefore, the evaluation of stiffness is far more crucial than that of the maximum fracture force.

Of all the surgical treatment options, K-wire fixation is the least invasive. Compared with bone plate fixation, K-wire fixation features a lower probability of soft tissue dissection and extensor tendon adhesion and offers a superior range of motion [21, 22]. However, the potential disadvantages of K-wire fixation include a poor ability to resist tilting deformation as well as the shortening of metacarpal bones. Other disadvantages include K-wire migration, pin tract infection, and a longer period of immobilization, which may delay rehabilitation [6, 8]. Despite causing soft tissue invasion, open reduction and internal fixation (ORIF) still has a role in treating patients with multiple concurrent metacarpal fractures, those who are unable to protect exposed pins, or who cannot tolerate a period of immobilization.

In our in vitro biomechanical experiment examining the fixation of metacarpal fractures using wires, the KW group had a maximum fracture force and stiffness of 94.0 + 17.4 N and 20.7 + 5.8 N/mm, respectively. We could not find studies featuring study conditions (i.e., artificial bones and metacarpal shaft) and testing methods (i.e., the cantilever bending test) similar to the current study for comparison. Among the existing studies, Jones et al. [23] used artificial bones identical to those in the current study. However, for similar fixation of metacarpal neck fractures, the maximum fracture force and stiffness they obtained were 279.7 + 100.3 N and 5.8 + 0.5 N/mm, respectively. For the fixation of metacarpal neck fractures using the KW method, the stiffness obtained by Chiu et al. [24] was 16.9 + 3 (median ± IQR), which is slightly greater than the stiffness of metacarpal neck fractures obtained in the current study. The most likely reason is that the artificial bones used for examining metacarpal neck fractures in these studies differed from those used in the current study, and the previous studies focused on metacarpal neck fractures rather than metacarpal shaft fractures. However, in real clinical application during surgery, the difficulty of fixating the metacarpal shaft is higher than that of fixating the metacarpal neck by using the KW method. This is because the fracture end is further from the K-wire entry point and has a more oblique trajectory. For a minimally invasive percutaneous insertion of K-wires through the skin, fixating them in the correct position is fairly difficult. When the K-wires are not placed in the correct location, the fixation strength of the method would become much weaker.

**Fig. 5:**
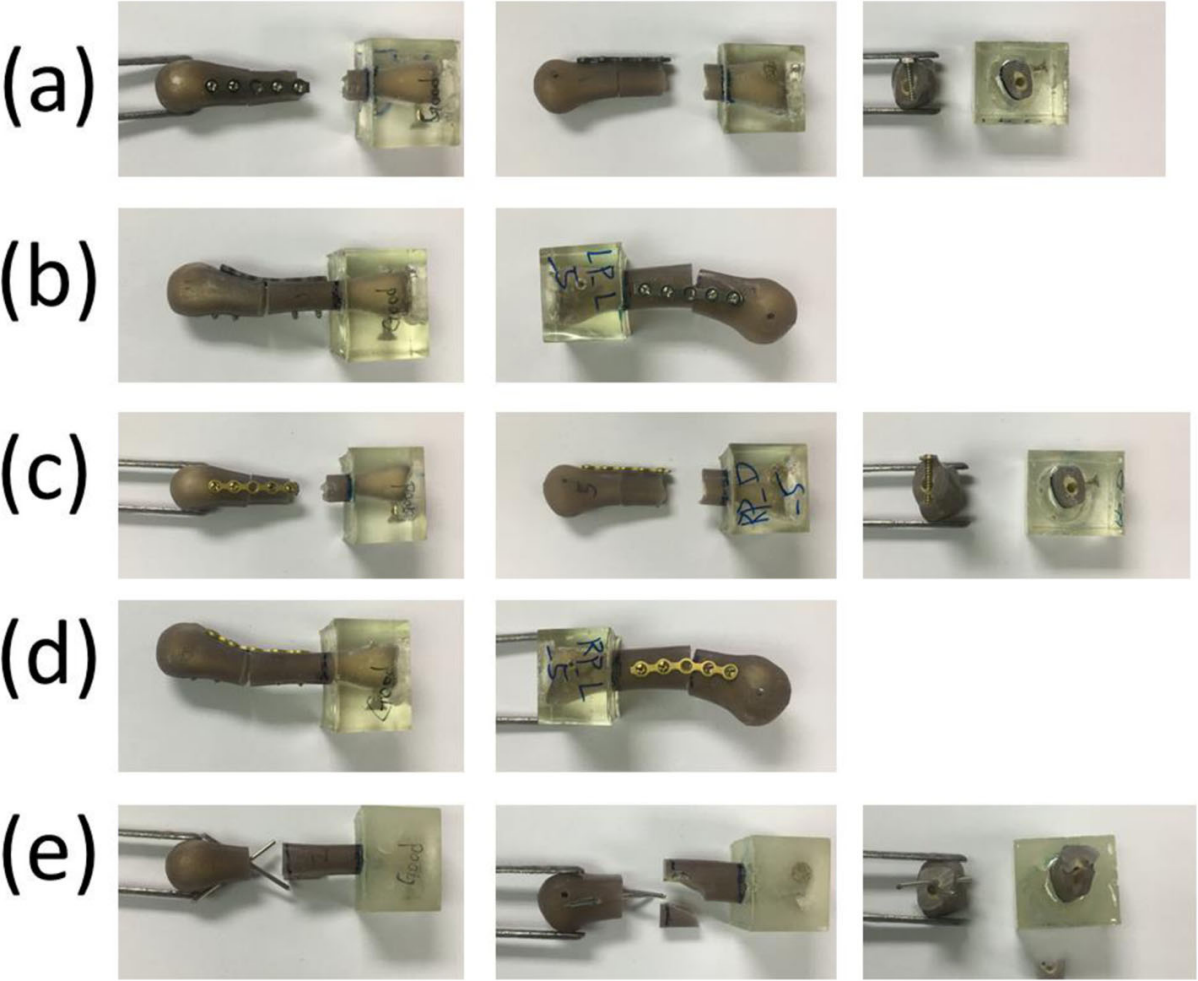
Failure modes of the five fixation approaches: (**a**) locking plate with four locking bicortical screws on the dorsal side; (**b**) locking plate with four locking bicortical screws on the lateral side; (**c**) regular plate with four regular bicortical screws on the dorsal side; (**d**) regular plate with four regular bicortical screws on the lateral side; and (**e**) two K-wires. Left column, anterior–posterior view; middle column, lateral view; right column, cross-section view

In recent years, the development of locking plates has markedly changed fracture treatment strategies; their strong fixation ability allows patients to begin their rehabilitation earlier, which in turn shortens the sick leave they require and allows better post-surgery joint movement angles to be obtained. However, a biomechanics study conducted by Doht et al. revealed that compared with a nonlocking plate, a locking plate did not offer superior fracture stability for metacarpal shaft fractures [25]. In the current study, the authors used bone plates for dorsal side fixation; two sets of experimental results indicated that no significant difference existed between the LP_D group (230.1 + 22.8N) and RP_D group (228.2 + 13.4N) in terms of the maximum fracture force. In terms of stiffness, that of the LP_D group (68.7 + 14.0N/mm) was approximately 25.1% higher than that of the RP_D group (54.9 + 3.2N/mm). From the clinical perspective, because the broken ends were all proximal to the plate, this indicated that when fixating metacarpal fractures by using dorsal bone plate fixation, both locking and nonlocking plates can offer sufficient fixation strength to resist the fracture deformation caused by hand motion; this is because people’s normal hand motion would not result in such tremendous bending force as applied in the experiment. Barr et al. [26] used artificial bones similar to those used in the current study as well as bending tests for testing the fixation ability of the locking plate and nonlocking plate in fixating metacarpal fractures. Similar to our study, their results indicated no statistical differences between the two. Furthermore, the maximum fracture force and stiffness values obtained by Barr et al. were slightly lower than those obtained in the current study; we inferred that this might be caused by the different brand of the bone plates and the different number of fixation screws used. Additionally, Tannenbaum et al. [27] similarly used locking plates for fixating metacarpal shaft fractures. The fracture force and stiffness values they obtained were 190 + 17 N and 11.2 + 1.0 N/mm, respectively. These values are slightly lower than those of the LP_D group in the current study. Apart from the different brands of locking plates, the differential results may also be caused by the fact that Tannenbaum et al. actually used cycle loading. With regard to the failure mode of bone plate usage, the results of the current study are identical to those of relevant studies; all the fracturing occurred near the proximal screw hole (Fig. 5).

In our pilot study, we measured the maximum fracture force and stiffness of the intact artificial metacarpal bones. The maximum fracture force (322.7 N) and stiffness (63.8 N/mm) of the intact artificial metacarpal bones were much higher than those of the lateral plating fixation approaches. Therefore, compared with lateral plating fixation, the dorsal plating approach provides more stable fixation.

In phalanges, lateral plating and dorsal plating offer similar mechanical strength (axial stiffness and loading to failure) and postoperative functioning [11, 28]. However, a retrospective study by Hustedt et al. [9] indicated that although lateral plating offers slightly better results for removing internal fixation and range of motion, the differences were not statistically significant [9]; the researchers were unable to identify the reason behind the higher failure rate of lateral side plating. Currently, no relevant literature exists on the effect of lateral plating on fixating metacarpal fractures. According to relevant studies and our past clinical experience, acceptable success rates can also be achieved with KW fixation for metacarpal shaft fractures [29]. In other words, if we treat the fixation strength of KW fixation as the minimum requirement for fixation strength, then the fixation strength that can be achieved through lateral placing of bone plates is quite worrisome. According to our research results, lateral plate fixation does not provide effective fixation effects for metacarpal fractures. Relevant studies have also indicated that lateral plate fixation would not lead to substantial enhancement of postoperative functioning. Therefore, fixation of metacarpal fractures using lateral plating is not recommended.

The current study has several limitations, which need to be explained before the conclusions are presented. First, the researchers used artificial bones for the experiments given that fresh and homogeneous human metacarpals are difficult to obtain. However, artificial bones are unable to fully simulate the characteristics of real human bones, which include inhomogeneity, anisotropy, and trabecular bone structure. Second, given the use of artificial bones, this study’s experimental samples did not contain soft tissues such as ligaments and tendons. Third, similar to relevant studies, the researchers used the cantilever bending testing method to test the fixation ability of different fracture fixation methods; however, this loading model still cannot fully simulate the actual condition of stress sustained by phalanges during movement. In addition, other fixation approaches (e.g., intramedullary wires, headless compression screws) require study in the future.

## Conclusion

Given the experimental setup and limitations of the current experimental study, our mechanics research results revealed that the mechanical strength offered by lateral plate fixation of metacarpal shaft fractures is so low that even KW fixation can offer relatively superior mechanical strength; this is regardless of whether a locking or nonlocking plate is used. Lateral plate fixation can reduce the probability of extensor tendon adhesion; nevertheless, the results of our mechanics research indicated that when lateral plate fixation is used for fixating metacarpal shaft fractures in a clinical setting, whether the mechanical strength offered would be strong enough to support bone union remains questionable.

## Data Availability

The data sets used and analyzed during the current study are available from the corresponding author upon reasonable request.

## Abbreviations

K-wire: Kirschner wire
LP_D: Locking plate with four locking bicortical screws on the dorsal side
LP_L: Locking plate with four locking bicortical screws on the lateral side
RP_D: Regular plate with four regular bicortical screws on the dorsal side
RP_L: Regular plate with four regular bicortical screws on the lateral side
KW: Two K-wires
